# TAZ Regulates the Cisplatin Resistance of Epithelial Ovarian Cancer Cells via the ANGPTL4/SOX2 Axis

**DOI:** 10.1155/2022/5632164

**Published:** 2022-09-22

**Authors:** Caihong Li, Qin Wang, Youzhen Luo, Juan Xiang

**Affiliations:** The First College of Clinical Medical Science, China Three Gorges University, Yichang City, 443000 Hubei Province, China

## Abstract

**Objective:**

Epithelial ovarian cancer (EOC) is a fatal gynecological malignancy. This study explored the mechanism of TAZ in regulating drug sensitivity of cisplatin (DDP-)-resistant EOC cells through the ANGPTL4/SOX2 axis.

**Methods:**

The A2780/DDP cells were prepared by stepwise progressive concentration method. The drug resistance and TAZ expression in EOC cells were determined. Drug sensitivity was measured after TAZ overexpression in A2780 cells and TAZ downregulation in A2780/DDP cells, respectively. The effects of TAZ knockdown on apoptosis rate, stemness, and cancer stem cell (CSC) marker (CD44, OCT4, and ALDH1A) levels in A2780/DDP and DDP-treated A2780/DDP cells were assessed. The binding of TAZ and ANGPTL4 was verified using ChIP-qPCR, and ANGPTL4 and SOX2 levels were determined. The effects of different combined treatments of TAZ, ANGPTL4, and SOX2 on drug sensitivity of A2780/DDP cells and DDP-treated A2780/DDP cells were evaluated.

**Results:**

TAZ was upregulated in drug-resistant EOC cells. TAZ knockdown significantly increased the drug sensitivity of A2780/DDP cells, while TAZ overexpression markedly decreased the drug sensitivity of A2780 cells. TAZ silencing promoted apoptosis of drug-resistant EOC cells and inhibited cell stemness. TAZ targeted ANGPTL4 and TAZ silencing enhanced drug sensitivity of A2780/DDP cells by inhibiting ANGPTL4. ANGPTL4 overexpression elevated SOX2 expression, and SOX2 downregulation reduced the drug resistance and promoted the apoptosis of A2780/DDP cells.

**Conclusion:**

TAZ regulates DDP sensitivity of drug-resistant EOC cells via the ANGPTL4/SOX2 axis.

## 1. Introduction

Epithelial ovarian cancer (EOC), generally recognized as the most lethal gynecological malignant tumor, is a heterogeneous disease that can be classified into the following five histological subtypes: mucinous carcinomas, clear cell carcinomas, endometrioid carcinomas, low-grade serous carcinomas, and serous carcinomas [1, 2]. EOC complicates up to 85-90% of all ovarian cancers and is commonly diagnosed at the advanced stage [3]. Every year, about 230,000 new cases arise, and EOC contributes to 150,000 deaths worldwide [4]. EOC has poor prognoses and low survival rates because of its high rate of recurrence, eventual chemoresistance, and diffuse dissemination [5]. Poly ADP-ribose polymerase inhibitors are recently authorized to initially treat EOC and play a role in the maintenance treatment [3]. Cytoreductive surgery assisted with chemotherapy utilizing cisplatin (DDP) blended with paclitaxel is an effective method in the initial treatment, while EOC will soon relapse and EOC patients even develop resistance to chemotherapy [6]. Therefore, understanding the drug resistance of EOC is essential for the development of new treatment therapies.

The Hippo pathway is an important signaling mechanism, which represses the growth of organs and cells during development and in disorders [7]. Transcriptional co-activator with PDZ-binding motif (TAZ) is a downstream effective factor of the Hippo pathway that can modulate tumorigenesis, tissue homeostasis, and organ size through functioning as a transcriptional co-activator [8]. Importantly, YAP/TAZ and Hippo pathways are imperative in tumorigenesis and migration of ovarian cancer cells [9]. Although the role of YAP is the first to be recognized, many studies also support the function of TAZ in ovarian cancer: for instance, elevated TAZ mRNA expression is associated with poor prognosis, and TAZ affects migration, proliferation, treatment response, and EMT of ovarian cancer [8, 10]. Moreover, in the intention study of Wen-Hsuan Yang et al., TAZ, rather than YAP, represents the dominant effector in the tested ovarian cancer cells [11]. Essentially, TAZ functionally participates in the migration and proliferation of EOC cells [12]. TAZ produces a major effect to promote the resistance to diverse anticancer therapies, such as radiation therapy, molecular targeted therapy, and cytotoxic chemotherapy [13, 14]. However, there are limited reports about the effect of TAZ on the drug resistance of EOC cells.

Angiopoetin-like 4 (ANGPTL4) is a member of the secreted protein family that has a similar structure to angiopoietin, which is imperative in various biological functions, such as vascular permeability, angiogenesis, chronic inflammation, and hematopoietic stem cell expansion [15, 16]. ANGPTL4 can combine with integrin *α*5*β*1 on the surface of ovarian cancer cells to induce drug resistance [17]. Sex determining region Y-box 2 (SOX2) promotes cancer cell proliferation and tumorigenesis and reduces apoptosis [18, 19]. ANGPTL4 overexpression can lead to the enrichment of glioma stem-like cells (GSCs), which is distinguished by the level of polycomb complex protein BMI-1 and SOX2 [20]. The amplification of SOX2 can be regarded as a potential biomarker for risk stratification and tumor development in EOC [21]. SOX2 is associated with the drug resistance of cancer stem cells (CSCs) [22]. Nonetheless, whether the ANGPTL4/SOX2 axis regulates drug resistance of EOC cells remains unknown. Therefore, this study sought to explore the mechanism of TAZ in modulating the drug resistance of EOC cells to provide a novel theoretical method for the clinical reversal of drug resistance in EOC.

## 2. Materials and Methods

### 2.1. Cell Culture and A2780/DDP Models

The human EOC cells (A2780) provided by the Shanghai cell bank of the Chinese Academy of Sciences (Shanghai, China) were cultured in the RPMI-1640 medium (Gibco, Carlsbad, CA, USA) containing 10% fetal bovine serum (Gibco) and 1% antibiotic (including 100 U/mL penicillin and 100 *μ*g/mL streptomycin) in a humidified incubator at 37°C with 5% CO_2_.

When the cell confluence reached 80%, *in vitro*DDP-resistant cells were prepared through the stepwise progressive concentration method [23]. Cells in the logarithmic growth phase were placed in culture flasks and then added with DDP (Sigma-Aldrich, St. Louis, MO, USA) at concentrations of 0.1, 0.2, 0.5, 1, and 2 *μ*g/mL, and treatments were repeated 5 times for each concentration. After 48 hours of cell culture, the medium was discarded, and cells were supplemented with fresh medium. After 12 months, the stable DDP-resistant cell line A2780/DDP was established at a concentration of 2 *μ*g/mL and cryopreserved in liquid nitrogen.

### 2.2. Cell Transfection

A2780/DDP cells were trypsinized, suspended in OPTI-minimal essential medium (Invitrogen, Carlsbad, CA, USA) at 5 × 10^4^ cells/mL, and then added to each well. Cell transfection was performed with Lipofectamine®2000 (Invitrogen) when the cell confluence exceeded 80%, with a final concentration of 50 nmol/L for each transfectant. Subsequent experiments were implemented after transfection for 48 hours.

A2780 cells were assigned into the following 10 groups: (1) A2780 group (DDP-sensitive cells); (2) A2780/DDP group (DDP-resistant cells); (3) A2780/DDP + siR-negative control (NC) group: siR-NC was transfected into A2780/DDP cells; (4) A2780/DDP + siR-TAZ group: siR-TAZ was delivered into A2780/DDP cells; (5) A2780 + pcDNA3.1-NC group: pcDNA3.1-NC was introduced into A2780 cells; (6) A2780 + pcDNA3.1-TAZ group: pcDNA3.1-TAZ was manipulated into A2780 cells; (7) A2780/DDP + siR-TAZ + oe-NC group: A2780/DDP cells were transfected with siR-TAZ and pcDNA3.1-NC; (8) A2780/DDP+siR-TAZ+oe-ANGPTL4 group: A2780/DDP cells were delivered with siR-TAZ and pcDNA3.1-ANGPTL4; (9) A2780/DDP + siR-TAZ + oe-ANGPTL4 + siR-NC group: A2780/DDP cells were introduced with siR-TAZ, pcDNA3.1-ANGPTL4 and siR-NC; (10) A2780/DDP + siR-TAZ + oe-ANGPTL4 + siR-SOX2 group: A2780/DDP cells were manipulated with siR-TAZ, pcDNA3.1-ANGPTL4 and siR-SOX2. siR-NC and siR-TAZ, pcDNA3.1-NC and pcDNA3.1-TAZ, pcDNA3.1-NC, and pcDNA3.1-ANGPTL4 were purchased from Sangon Biotech (Shanghai, China). siR-NC and siR-SOX2 were purchased from SunBio (Shanghai, China). The sequences were as follows: siR-TAZ, 5′-CCCUAGGAAGGCGAUGAAUTT-3′; siR-SOX2, 5′-CCUGUGGUUACCUCUUCCCCCACU-3′; and siR-NC, 5′-UUCUCCGAACGUGUCACGUTT-3′.

### 2.3. Cell Counting Kit-8 (CCK-8)

CCK-8 kit (KeyGen Biotech Co. Ltd., Nanjing, China) was employed to detect the resistance of A2780 and A2780/DDP cells to DDP. Briefly, A2780 cells or A2780/DDP cells were collected and seeded in 96-well plates at 1 × 10^5^ cells/mL and 100 *μ*L/well, respectively. Cells adhered overnight and were treated with DDP at concentrations of 0, 15, 30, 60, 120, and 240 nM for 48 hours. Afterwards, cells were added with 10 *μ*L CCK-8 per well and incubated for 1 hour at 37°C. Later, the optical density value at 450 nm was measured.

### 2.4. Flow Cytometry

Annexin-V/fluorescein isothiocyanate (FITC) kit (556547, BD Biosciences, San Jose, CA, USA) was applied for apoptosis detection. The cells were seeded into 6-well plates at 2 × 10^5^ cells/well. After 24 hours, the culture medium was replaced with the DDP-containing medium and incubated for 48 hours under appropriate conditions. After centrifugation, the cells were washed twice with phosphate buffer saline (PBS, 1 ×), stained with Annexin-V/FITC and propidium iodide (PI), and then incubated at room temperature for 15 minutes under dark conditions. Annexin-V was used to determine the content of phosphatidylserine during apoptosis, and PI was adopted to differentiate necrotic cells from healthy cells. The apoptosis rate was examined by the flow cytometer (BD Biosciences).

### 2.5. Reverse Transcription Quantitative Polymerase Chain Reaction (RT-qPCR)

When the cell confluence reached 80-90%, the TRIzol reagent (Invitrogen, Carlsbad, CA, USA) was added to lyse the cells and extract total RNA. RNA was reversely transcribed into cDNA using a PrimeScript™RT Master Mix kit (Takara, Tokyo, Japan). The qPCR was performed using the ABI VII7 PCR detection system (ABI, Foster City, CA, USA). The cycling conditions were as follows: initial predenaturation at 95°C for 30 s, and then, 40 PCR cycles of denaturation at 95°C for 5 s, annealing at 60°C for 34 s, and final annealing at 95°C for 15 s, 60°C for 60 s, and extension at 95°C for 15 s. GAPDH served as the endogenous control, and the 2^-*ΔΔ*CT^ method was employed to calculate the relative mRNA levels. The qPCR primer sequences are shown in Table 1.

### 2.6. Western Blot (WB)

After transfection for 48 hours, the cell protein lysates were extracted with radioimmunoprecipitation assay (RIPA) buffer (Beyotime, Shanghai, China) according to the provided instructions. Proteins were separated by 8% sodium dodecyl sulfate (SDS-)-polyacrylamide gel electrophoresis and transferred to polyvinylidene fluoride membranes. The membranes were then blocked with 5% nonfat dry milk for 1.5 hours at 4°C and rinsed 3 times with Tris-buffered saline with Tween (TBST) for 10 minutes each time. Subsequently, the samples were incubated overnight with primary antibodies against TAZ (1 : 1000, ab119373, Abcam, Cambridge, MA, USA), ANGPTL4 (1 : 1000, ab196746, Abcam), SOX2 (1 : 1000, ab137385, Abcam), CD44 (1 : 2000, ab157107, Abcam), OCT4 (1 : 1000, ab137427, Abcam), and ALDH1A1 (1 : 5000, ab227964, Abcam) at 4°C. Later, the samples were incubated with diluted corresponding secondary antibody IgG (1 : 2000, ab6721, Abcam). After 3 TBST rinsing, the membranes were visualized using enhanced chemiluminescence solution, exposed to X-ray, developed, and fixed, and then, the gray values of target bands were analyzed with gel image processing system software (UVP, Inc., Upland, CA, USA). GAPDH (1 : 2000, ab245355, Abcam) was used as an internal reference.

### 2.7. Spheroid Formation Assay

The individual cells (10^4^ cells/mL) were cultured in serum-free Dulbecco's modified Eagle medium/F-12 containing B27 (Invitrogen), 20 ng/mL epidermal growth factor (BD Bioscience), and 10 ng/mL basic fibroblast growth factor (BD Bioscience). After culture for 7 to 10 days in the ultra-low adherent plate (Corning, NY, USA) under conventional conditions, the spheres were photographed, and tumor spheres larger than 50 *μ*m in diameter were counted.

### 2.8. Chromatin Immunoprecipitation (ChIP) Analysis

ChIP-qPCR experiments were performed according to the Myers Lab ChIP-seq protocol [24]. Briefly, A2780 cells were incubated in the crosslinking solution (containing 1% formaldehyde) for 10 minutes at room temperature, and then, crosslinking was terminated by adding glycine at 0.125 M. Cells were rinsed with cold PBS and suspended in Farnham lysis buffer (containing 5 mM 1,4-piperazinediethanesulfonic acid with pH 8.0, 85 mM KCI, and 0.5% NP-40) newly supplemented with protease inhibitors. The lysate was punctured 20 times by a 20G needle to rupture the cells and keep the nuclei intact. After centrifugation, cells were resuspended with RIPA buffer newly supplemented with protease inhibitors. Chromatin was fragmented using Bioruptor (Diagenode, Liège, Belgium) high-speed sonication for 30 minutes (30 seconds ON, 30 seconds OFF). Proteins were immunoprecipitated in PBS/bovine serum albumin buffer using TAZ antibody (Cell Signal, #70148) or control antibody, rabbit IgG (Cell Signal, #2729), and these antibodies had been coupled to dynabeadstm protein G magnetic beads (Thermo Fisher Scientific #10004D, Waltham, MA, USA) for 2 hours at 4°C. Next, the antibody-chromatin complexes were washed 5 times with lithium chloride solution, followed by TE solution. Samples were eluted overnight with the elution buffer (containing 1% SDS, 0.1 M NaHCO_3_) at 65°C. The eluted product was purified with the QIAquick PCR purification kit (QIAGEN #28104, Dusseldorf, Germany) and subsequently used for ChIP analysis. The CTGF promoter served as a positive control for the target genes of TAZ, with Ch10 as a negative control [25]. The primer sequences are exhibited in Table 2.

### 2.9. Enzyme-Linked Immunosorbent Assay (ELISA)

The collected cell culture supernatant was centrifuged and then transferred to clean tubes. The level of ANGPTL4 in cell culture supernatant was determined using human ANGPTL4 ELISA kits (RayBiotech, Norcross, GA, USA). Following the kit manufacturer's instructions, the concentration was determined, normalized to the cell number, and expressed as pg/10^5^ cells. All experiments were conducted at least thrice.

### 2.10. Statistical Analysis

The data assay and mapping were implemented using SPSS 21.0 statistical software (IBM Corp. Armonk, NY, USA) and GraphPad Prism 8.01 software (GraphPad Software Inc., San Diego, CA, USA). The data were exhibited as mean ± standard deviation (SD). The *t* test was employed for comparisons between two groups. The one-way analysis of variance (ANOVA) was adopted for comparisons among multiple groups. Tukey's multiple comparisons test was applied for the post hoc analysis. The *P* value <0.05 indicated statistical significance.

## 3. Results

### 3.1. TAZ Was Upregulated in Drug-Resistant EOC Cells

**Figure 1:**
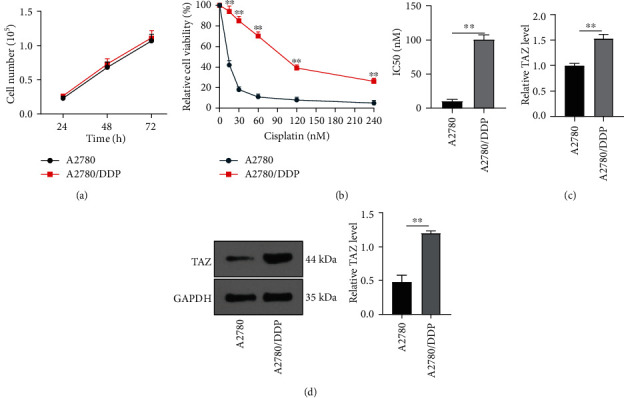
TAZ was highly expressed in drug-resistant EOC cells. The A2780/DDP cell models were established. (a) Cell viability was detected by CCK-8. (b) After EOC cells were treated with cisplatin at different concentrations for 48 hours, the drug sensitivity of EOC cells was detected by CCK-8. (c) TAZ mRNA expression was determined using RT-qPCR. (d) Protein expression of TAZ was measured via WB. Cell experiment was repeated three times. Data were expressed as mean ± SD, and independent sample *t* test was used for comparisons between the two groups. ^∗∗^*P* < 0.01.

To study the effect of TAZ on the DDP sensitivity of drug-resistant EOC cells, the A2780/DDP cell models were first established. CCK-8 assay showed that the growth rate of A2780 was similar to that of A2780/DDP cells (*P* > 0.05) and the doubling time of A2780/DDP cells was the same as that of A2780 cells (*P* > 0.05) (Figure 1(a)). In addition, after EOC cells were treated with different concentrations of DDP for 48 hours, we observed that the cell viability of the A2780/DDP group was clearly increased compared with the A2780 group (*P* < 0.01) (Figure 1(b)), and drug resistance was enhanced. Moreover, the IC50 of DDP in the A2780/DDP group was significantly higher than that of the A2780 group (*P* < 0.01) (Figure 1(b)), indicating that A2780 cells were more sensitive to DDP than A2780/DDP cells. Subsequently, RT-qPCR and WB indicated markedly upregulated TAZ levels in the A2780/DDP group relative to the A2780 group (*P* < 0.01) (Figures 1(c) and 1(d)). Briefly, TAZ was highly expressed in drug-resistant EOC cells, which might be related to drug resistance in EOC cells.

### 3.2. Knockdown of TAZ Improved the Drug Sensitivity of DDP-Resistant EOC Cells

**Figure 2:**
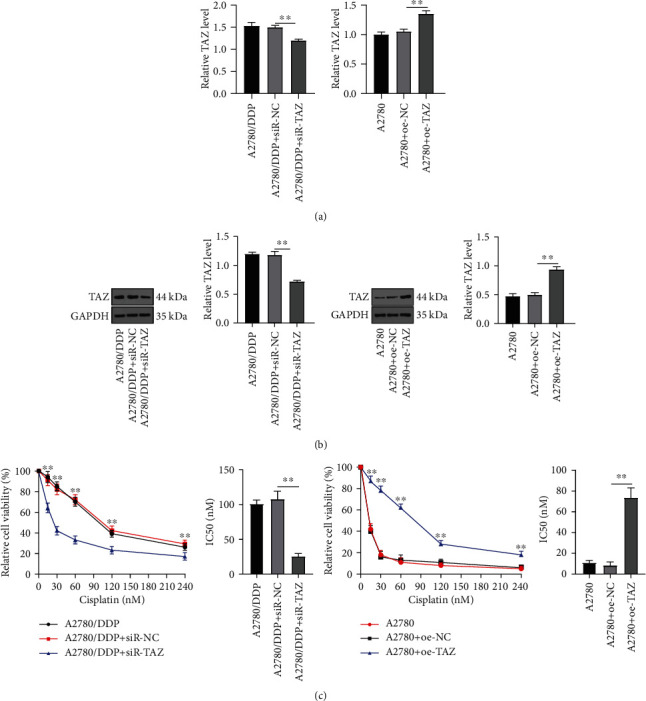
Knockdown of TAZ improved the drug sensitivity of EOC cells. Drug-resistant EOC cells and EOC cells were transfected with siR-TAZ and pcDNA3.1-TAZ, respectively. (a) RT-qPCR was used to detect the expression of TAZ. (b) WB was employed to determine the protein expression of TAZ. (c) After cells were treated with different concentrations of cisplatin for 48 hours based on transfection with siR-TAZ and pcDNA3.1-TAZ, CCK-8 was employed to measure the drug sensitivity of EOC cells. Cell experiment was repeated three times. Data were shown as mean ± SD, and one-way ANOVA was adopted for comparisons among groups. Tukey's multiple comparisons test was implemented for the post hoc test. ^∗^*P* < 0.05, ^∗∗^*P* < 0.01.

Knockdown of TAZ leads to the reduced proliferation of ovarian cancer cells, and the verteporfin, a complex that disrupts the interaction between YAP/TAZ and TEAD, can reduce ovarian cancer cell viability [26]. Moreover, the previous results had unveiled upregulated TAZ levels in drug-resistant EOC cells. Therefore, to explore the impact of TAZ on the DDP sensitivity of drug-resistant EOC cells, the cells were transfected with siR-TAZ to knock down the TAZ in drug-resistant EOC cells. RT-qPCR and WB demonstrated remarkably decreased TAZ levels in the A2780/DDP + siR-TAZ group compared with the A2780/DDP + siR-NC group (*P* < 0.01) (Figures 2(a) and 2(b)). Later, TAZ was overexpressed by transfection of pcDNA3.1-TAZ into EOC cells, and the findings suggested that TAZ in the A2780 + oe-TAZ group was prominently elevated relative to that in the A2780 + oe-NC group (*P* < 0.05) (Figures 2(a) and 2(b)). In addition, the drug sensitivity of EOC cells was examined by CCK-8 assay. The results manifested that after the knockdown of TAZ, the drug sensitivity of A2780/DDP cells was increased, while TAZ overexpression significantly reduced the drug sensitivity of A2780 cells (all *P* < 0.01) (Figure 2(c)). In short, TAZ was associated with DDP resistance in EOC cells, and inhibition of TAZ enhanced the DDP sensitivity of drug-resistant EOC cells.

### 3.3. Knockdown of TAZ Promoted Drug-Resistant EOC Cell Apoptosis and Inhibited Cell Stemness

**Figure 3:**
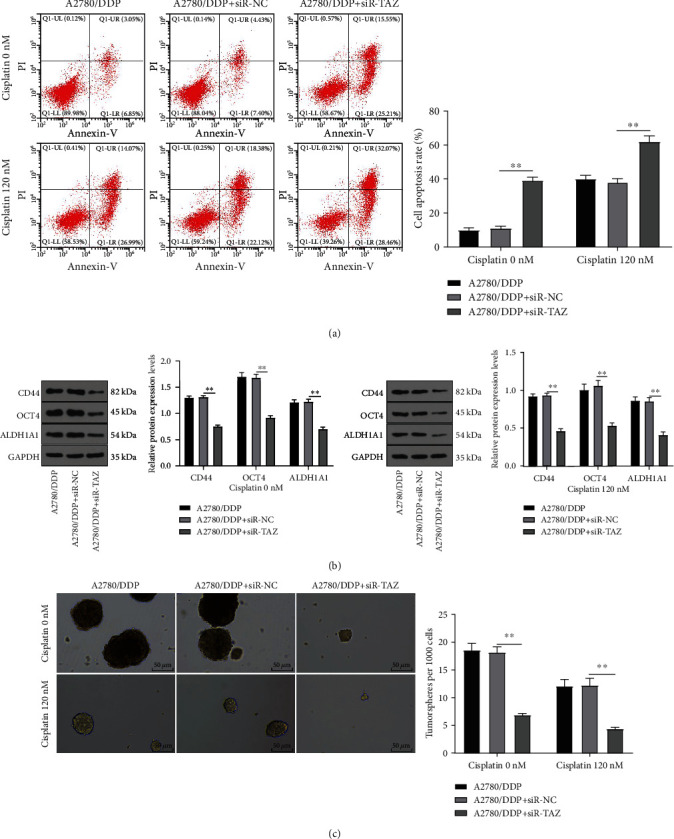
Knockdown of TAZ inhibited the drug-resistant EOC cell stemness. The drug-resistant EOC cells were transfected with siR-TAZ or treated with 120 nM cisplatin for 48 hours. (a) Apoptosis rate was measured by flow cytometry. (b) The expression of biomarkers for CSCs was detected by WB. (c) Impairment of tumorsphere formation was observed by spheroid formation assay. Cell experiment was repeated three times. Data were presented as mean ± SD, and one-way ANOVA was used for comparisons among groups. Tukey's multiple comparisons test was employed for the post hoc test. ^∗∗^*P* < 0.01.

Accumulating studies have reported that changes in cancer cell stemness affect the drug resistance of cancer cells [27–29]. To verify whether TAZ can affect the drug-resistant EOC cell stemness, A2780/DDP and 120 nM (IC50) DDP-treated A2780/DDP cells were selected for further experiments. Firstly, flow cytometry showed that knockdown of TAZ markedly increased the apoptosis rate of both the A2780/DDP and DDP-treated A2780/DDP group compared with the A2780/DDP + siR-NC group (*P* < 0.01) (Figure 3(a)). WB detection unveiled that TAZ knockdown brought about remarkable downregulation of some CSCs markers (including CD44, OCT4, and ALDH1A) (*P* < 0.01) (Figure 3(b)). Meanwhile, the spheroid formation assay revealed a decreased sphere-forming ability after TAZ knockdown (*P* < 0.05) (Figure 3(c)). Altogether, TAZ knockdown promoted apoptosis and restrained the stemness of drug-resistant EOC cells.

### 3.4. ANGPTL4 Was a Direct Target Gene of TAZ to Regulate Cell Resistance

**Figure 4:**
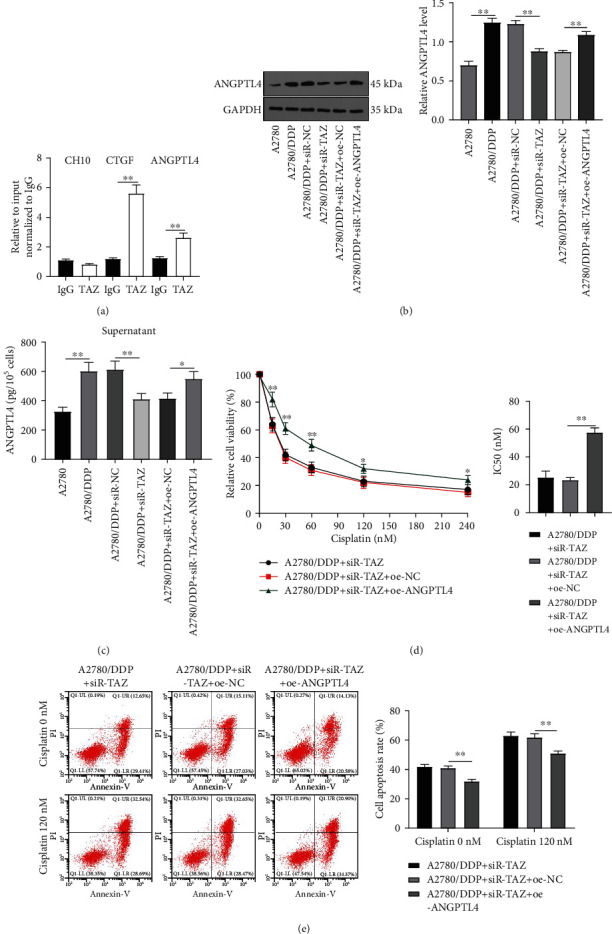
ANGPTL4 was a direct target gene of TAZ to regulate cell resistance. (a) ChIP-qPCR was used to verify the binding relationship of TAZ and ANGPTL4. (b) WB was adopted to detect the protein expression of ANGPTL4. (c) ELISA was used to determine the expression of ANGPTL4 in cell supernatant. (d) CCK-8 was employed to detect the drug sensitivity of drug-resistant EOC cells. (e) Flow cytometry was implemented to measure the apoptosis rate. Cell experiment was repeated three times. Data were expressed as mean ± SD, and one-way ANOVA analysis was employed for comparisons among groups. Tukey's multiple comparisons test was used for the post hoc test. ^∗^*P* < 0.05, ^∗∗^*P* < 0.01.

Overexpression of ANGPTL4 is capable of inducing the enrichment of GSCs and leads to the resistance of glioma cells to temozolomide [20]. The significant correlation between ANGPTL4 and TAZ expression is unveiled by the TCGA ovarian cancer dataset, and the regulatory region of ANGPTL4 is physically associated with the YAP/TAZ/TEAD complex [25, 30]. At first, ChIP-qPCR was adopted to validate the binding relationship between TAZ and ANGPTL4, and the results suggested that the promoter region of ANGPTL4 was similar to that of CTGF (positive control) and was enriched in the TAZ pull-down region (Figure 4(a)), illustrating the correlation of ANGPTL4 promoter and TAZ. Subsequently, WB and ELISA found that ANGPTL4 levels in cell lysate and cell supernatant were upregulated in A2780/DDP cells compared with A2780 cells, while inhibition of TAZ markedly decreased ANGPTL4 levels (all *P* < 0.01) (Figures 4(b) and 4(c)).

To further identify whether TAZ could affect the sensitivity of A2780/DDP cells to DDP by targeting ANGPTL4, ANGPTL4 overexpression was achieved in TAZ-downregulated cells. WB and ELISA revealed that the A2780/DDP + siR-TAZ + oe-ANGPTL4 group exhibited higher ANGPTL4 levels in the cell lysate and supernatant than the A2780/DDP + siR-TAZ + oe-NC group (*P* < 0.05) (Figures 4(b) and 4(c)). Afterwards, the drug sensitivity of drug-resistant EOC cells was determined by CCK-8 assay. The results revealed that ANGPTL4 overexpression partially annulled the repressive effect of TAZ silencing on the drug resistance of the A2780/DDP group in comparison with the A2780/DDP + siR-TAZ + oe-NC group (*P* < 0.05) (Figure 4(d)). Meanwhile, the effect of ANGPTL4 overexpression on the apoptosis rate of drug-resistant cells was assessed, and the results suggested that ANGPTL4 overexpression partially averted the promoting effect of TAZ silencing on the apoptosis rate of A2780/DDP and DDP-treated A2780/DDP cells (*P* < 0.01) (Figure 4(e)). Altogether, the knockdown of TAZ reduced the stemness of drug-resistant EOC cells and improved the drug sensitivity by targeting ANGPTL4.

### 3.5. Knockdown of TAZ Increased the Drug Sensitivity of Drug-Resistant EOC Cells through ANGPTL4/SOX2

**Figure 5:**
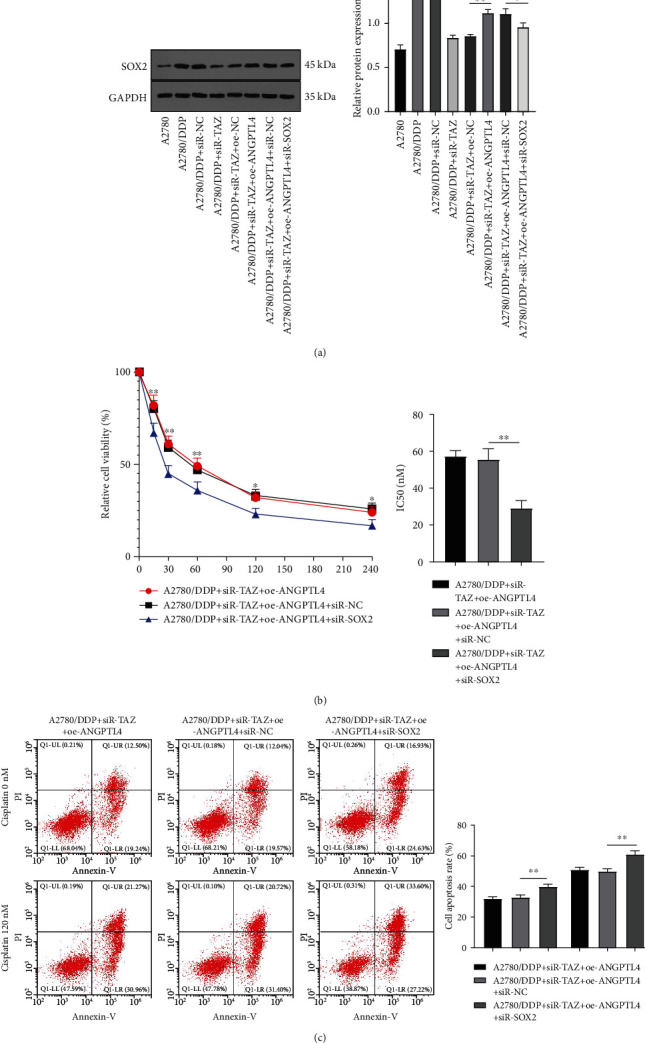
ANGPTL4 regulated the DDP sensitivity of drug-resistant EOC cells through SOX2. The drug-resistant EOC cells were transfected with siR-SOX2 based on TAZ knockdown and ANGPTL4 overexpression. (a) Protein expression of SOX2 was detected by WB. (b) Drug sensitivity was measured by CCK-8. (c) Apoptosis rate was determined by flow cytometry. Cell experiment was repeated three times. Data are presented as mean ± SD and one-way ANOVA was used for comparisons among groups. Tukey's multiple comparisons test was adopted for the post hoc test. ^∗^*P* < 0.05, ^∗∗^*P* < 0.01.

ANGPTL4 overexpression can induce the GSCs enrichment, which is characterized by the expression of polycomb complex proteins BMI-1 and SOX2, contributing to the resistance of glioma cells to temozolomide [20]. SOX2 is vital in regulating the drug resistance of cells [31]. WB assay showed that SOX2 was significantly increased in A2780/DDP cells compared with A2780 cells, whereas inhibition of TAZ markedly decreased SOX2, and ANGPTL4 overexpression partially reversed SOX2 (all *P* < 0.01) (Figure 5(a)). To further explore whether ANGPTL4 regulates the drug resistance of EOC cells via SOX2, cells in the A2780/DDP + siR-TAZ + oe-ANGPTL4 group were transfected with siR-SOX2 to perform a combined experiment. WB results revealed that SOX2 in the combined treatment group was remarkably reduced relative to the A2780/DDP + siR-TAZ + oe-ANGPTL4 + siR-NC group (*P* < 0.05) (Figure 5(a)). Subsequently, the changes in drug resistance and apoptosis rate of drug-resistant cells were determined, and the results unveiled that SOX2 knockdown diminished the DDP resistance (*P* < 0.05) (Figure 5(b)) and increased the apoptosis rate (all *P* < 0.01) (Figure 5(c)) of cells in the A2780/DDP + siR-TAZ + oe-ANGPTL4 group. Briefly, ANGPTL4 regulated the DDP sensitivity of drug-resistant EOC cells through SOX2.

## 4. Discussion

EOC is one of the commonly diagnosed malignancies among women worldwide and a chief cause of gynecologic cancer death [4]. In clinical data, drug resistance is a primary contributor to the poor prognosis in EOC patients [32]. TAZ can raise the resistance to different anticancer treatments, such as cytotoxic chemotherapy [33]. Therefore, this study explored the mechanism of TAZ in DDP resistance of EOC cells to provide a reference value for therapy and prognosis improvement.

TAZ, as an important member of the Hippo pathway, plays pivotal roles in drug resistance and tumorigenesis [34]. Preceding evidence supports that TAZ is upregulated in patients with ovarian cancer and TAZ overexpression facilitates the proliferation and migration of ovarian cancer cells [26]. Herein, we detected the expression of TAZ in A2780/DDP cells. Firstly, the A2780/DDP cells were prepared, and elevated TAZ was measured in drug-resistant EOC cells. A previous study has reported the regulation of TAZ on chemoresistance in EOC [11]. Subsequently, the A2780/DDP cells were transfected with siR-TAZ, and A2780 cells were delivered with pcDNA3.1-TAZ to explore the role of TAZ in the drug resistance of EOC cells. Our studies unveiled that TAZ silencing enhanced the drug sensitivity of A2780/DDP cells, whereas TAZ overexpression decreased the drug sensitivity of A2780 cells. Nagashima et al. also found that TAZ upregulation raises the drug resistance in cancer cells and TAZ inhibitor is beneficial for cancer treatment [35], indicating that TAZ is associated with DDP resistance of EOC cells, and inhibition of TAZ could significantly increase the drug sensitivity of the drug-resistant EOC cells. TAZ downregulation is a crucial mechanism in chemotherapy resistance [14].

An existing study reveals that the stemness of cancer cells is responsible for drug resistance, distal metastasis, and cancer relapse [36]. Therefore, we determined the effect of TAZ on cell apoptosis and stemness of A2780/DDP and DDP-treated A2780/DDP cells. Our findings noted that after TAZ knockdown, the apoptosis rate was increased, and CSC markers (CD44, OCT4, and ALDH1A) were downregulated; additionally, the tumor sphere formation ability was decreased significantly. Relevant research has also documented that TAZ may induce tumor stem cell proliferation, ultimately leading to metastasis and drug resistance in cervical cancer [37], while TAZ silencing weakens the viability of cervical cancer cells [38]. Furthermore, tumor sphere formation and CSC maintenance can be induced by TAZ [39, 40]. Collectively, TAZ knockdown boosted the drug-resistant EOC cell apoptosis and suppressed the stemness.

ANGPTL4 plays an essential role in the carboplatin resistance of ovarian cancer [17]. Hence, we investigated the relationship between ANGPTL4 and TAZ in drug-resistant EOC cells. ChIP-qPCR verified that the promoter region of ANGPTL4 was similar to that of CTGF (positive control) and enriched in the TAZ pull-down region, indicating the correlation of ANGPTL4 promoter and TAZ. WB assay suggested an elevated ANGPTL4 expression in A2780/DDP cells but diminished ANGPTL4 expression after TAZ downregulation. Consistently, ANGPTL4 is upregulated in EOC cells [41]. The cooperation of ANGPTL4 and TAZ has been documented in chemoresistance in EOC [11]. Subsequently, we explored the role of ANGPTL4 in the drug resistance of EOC cells. The results showed that ANGPTL4 overexpression partly annulled the inhibition of TAZ silencing on DDP resistance of A2780/DDP cells and the promotion of TAZ silencing on the apoptosis rate of A2780/DDP and DDP-treated A2780/DDP cells. The induction of drug resistance by ANGPTL4 has been reported in ovarian cancer [17]. ANGPTL4 is one of the protumorigenic proteins which can stimulate cancer cell growth and promote cancer metastasis [42]. ANGPTL4 upregulation parallels the elevated expression of stem cells markers [43]. Altogether, TAZ silencing could reduce A2780/DDP cell stemness and enhance drug sensitivity by targeting ANGPTL4.

ANGPTL4 is involved in the regulation of tumorigenesis and stem cells [44], and SOX2 is a major stem cell marker [45]. Therefore, we investigated the correlation between ANGPTL4 and SOX2. WB results indicated that SOX2 was upregulated in A2780/DDP cells, while TAZ knockdown decreased SOX2 expression, and ANGPTL4 overexpression partly restored SOX2 expression. SOX2 is elevated in ovarian cancer cells [46]. TAZ silencing can decrease the SOX2 at the levels of mRNA and protein [40]. SOX2 overexpression is regarded as a marker for taxane resistance in stage III EOC patients [47]. To explore the effect of SOX2 on drug resistance of EOC cells, the cells were delivered with siR-SOX2 to perform the combined experiments. After SOX2 downregulation, the drug resistance was reduced, and the apoptosis rate was increased in drug-resistant EOC cells. As a crucial transcription factor, SOX2 can trigger drug resistance in cancer cells [48]. SOX2 knockdown effectively inhibits the antiapoptotic gene expression and drug resistance and enhances the DDP sensitivity of oral cancer cells [49]. The aforementioned results unraveled that ANGPTL4 modulated the drug sensitivity of drug-resistant EOC cells through SOX2.

In conclusion, this study confirmed that TAZ regulated the drug resistance of EOC cells via the ANGPTL4/SOX2 axis. However, there are several limitations: (1) The results lacked *in vivo* validation in animal experiments and clinical data; (2) we only validated the relationship between ANGPTL4 (a downstream target of TAZ) and drug resistance of EOC cells, and other downstream targets of TAZ warrant to be further explored; (3) the specific mechanism of TAZ in the ANGPTL4/SOX2 pathway needs to be explored in depth; and (4) we only used one siRNA-TAZ in this study. In the future, we shall examine additional siRNA to confirm on target effects, and carry out *in vivo* experiments and clinical studies on drug-resistant EOC cells, and explore the mechanism of TAZ in regulating other downstream target genes. Moreover, other signaling pathways that regulate drug resistance in EOC cells can be investigated.

## Figures and Tables

**Table 1:** Primer sequences.

Gene	Forward 5′-3′	Reverse 5′-3′
TAZ	ATTCATCGCCTTCCTAGGGT	GGCTGGGAGATGACCTTCAC
ANGPTL4	GGCTCAGTGGACTTCAACCG	CCGTGATGCTATGCACCTTCT
GAPDH	GTCATCCAACGGGAATGCA	TGATCGGTTACCGTGATCAAAA

Note: TAZ: transcriptional co-activator with PDZ-binding motif; ANGPTL4: angiopoietin-like 4; GAPDH: glyceraldehyde-3-phosphate dehydrogenase.

**Table 2:** Primer sequences.

Gene	Forward 5′-3′	Reverse 5′-3′
CTGF	GCCAATGAGCTGAATGGAGT	CAATCCGGTGTGAGTTGATG
ANGPTL4	GTCTCCCACGGTTCGTAGAG	TATAAGTTGGGTGCGGAGTGG
Ch10	ACCAACACTCTTCCCTCAGC	TTATTTTGGTTCAGGTGGTTGA

Note: CTGF: connective tissue growth factor; ANGPTL4: angiopoietin-like 4.

## Data Availability

All the data generated or analyzed during this study are included in this published article.
